# Bagging Improves the Performance of Deep Learning-Based Semantic Segmentation with Limited Labeled Images: A Case Study of Crop Segmentation for High-Throughput Plant Phenotyping

**DOI:** 10.3390/s24113420

**Published:** 2024-05-26

**Authors:** Yinglun Zhan, Yuzhen Zhou, Geng Bai, Yufeng Ge

**Affiliations:** 1 Department of Statistics, University of Nebraska-Lincoln, Lincoln, NE 68583, USA; 2 Department of Biological Systems Engineering, University of Nebraska-Lincoln, Lincoln, NE 68583, USA; 3 Center for Plant Science Innovation, University of Nebraska-Lincoln, Lincoln, NE 68583, USA

**Keywords:** field-based high-throughput plant phenotyping (FHTPP), high-resolution RGB image, semantic segmentation, deep learning, bagging

## Abstract

Advancements in imaging, computer vision, and automation have revolutionized various fields, including field-based high-throughput plant phenotyping (FHTPP). This integration allows for the rapid and accurate measurement of plant traits. Deep Convolutional Neural Networks (DCNNs) have emerged as a powerful tool in FHTPP, particularly in crop segmentation—identifying crops from the background—crucial for trait analysis. However, the effectiveness of DCNNs often hinges on the availability of large, labeled datasets, which poses a challenge due to the high cost of labeling. In this study, a deep learning with bagging approach is introduced to enhance crop segmentation using high-resolution RGB images, tested on the NU-Spidercam dataset from maize plots. The proposed method outperforms traditional machine learning and deep learning models in prediction accuracy and speed. Remarkably, it achieves up to 40% higher Intersection-over-Union (IoU) than the threshold method and 11% over conventional machine learning, with significantly faster prediction times and manageable training duration. Crucially, it demonstrates that even small labeled datasets can yield high accuracy in semantic segmentation. This approach not only proves effective for FHTPP but also suggests potential for broader application in remote sensing, offering a scalable solution to semantic segmentation challenges. This paper is accompanied by publicly available source code.

## 1. Introduction

Plant phenotyping is a crucial component of precision agriculture, as it allows crop breeders to gather detailed information about the growth and health of their crops. By measuring various traits such as plant size, height, and photosynthetic efficiency, scientists can optimize yields, detect disease, and improve sustainability. In recent years, advancements in imaging and sensing technologies have led to the development of non-invasive, digital plant phenotyping methods [1,2]. Compared to manual measurement, high-throughput plant phenotyping (HTPP) is more efficient and less labor-intensive to gather large amounts of data quickly and easily [3]. Moreover, sophisticated image analysis algorithms and machine learning techniques can help extract meaningful insights from the data, providing farmers with actionable information about their crops, such as identifying disease outbreaks, monitoring growth conditions, and optimizing fertilizer and irrigation use [2]. Crop segmentation, which separates the crop pixels from background ones (e.g., soil and weed) is often an initial and vital step in the image processing protocols of HTPP studies (e.g., estimation of canopy coverage rate, aboveground biomass, and leaf area index). This segmentation is typically achieved using a type of computer vision algorithm known as semantic segmentation, which assigns every pixel in an image to a categorical class label [4,5,6,7].

The history of semantic segmentation dates back several decades to early work in computer vision [8]. During the early stage, researchers may use a variety of low-level features that are extracted from the texture and color of images to perform segmentation tasks. For example, pixel color, Histogram of Oriented Gradients (HOG) [9], Scale Invariant Feature Transform (SIFT) [10], SURF [11], Features from Accelerated Segment Test (FAST) [12], FAST- ER [13], AGAST [14] and Multiscale AGAST [15] Detector, and Textons [16]. The methods used include Watersheds [17], clustering [18], and threshold [19]. However, these are unsupervised methods that may not give satisfying segmentation results for difficult tasks [20]. To overcome this, supervised machine learning models have been applied to pixel-wise image classification, such as support vector machine (SVM) [21], random forest (RF) [22], variations of Markov Random Field, and Conditional Random Field (CRF) [23,24]. Recently, researchers have developed various Deep Convolutional Neural Network (DCNN) architectures for semantic segmentation. These deep learning models yield remarkable performance improvements that often achieve the highest accuracy rates on popular benchmarks [25]. A breakthrough in deep learning semantic segmentation was made by proposing the Fully Convolutional Network (FCN) [26]. It adopted well-known classification nets (AlexNet [27], the VGG Net [28], and GoogLeNet [29]) by adding skip architecture and replacing the fully connected layers with convolutional layers, which was followed by upsampling layers to recover the spatial resolution and produce the per-pixel classification results. Ronneberger et al. proposed the U-Net architecture for biomedical image segmentation [30]. They used a compression path (encoder) to extract the context of input images and an expanding path (decoder) to restore the spatial information and produce pixel classification results. They also added skip-connections to concatenate contracting and expanding convolutional layers. Similarly, Badrinarayanan et al. developed a DCNN structure called Segnet [31]. The decoder of Segnet requires less resource by using max pooling indices from the encoder to perform upsampling. A sequence of DCNN structures called Deeplab for semantic segmentation was developed [32,33,34,35]. It introduced atrous convolution that can effectively enlarge the field of view without increasing the number of parameters or the amount of computation, and Atrous Spatial Pyramid Pooling (ASPP), which is capable of extracting multiscale features.

Semantic segmentation algorithms have significantly advanced over the years, providing powerful tools for image analysis. However, despite their historical significance and continued development, accurately and timely achieving crop segmentation for images captured under field conditions remains a challenge due to varying environmental illumination, weather and soil conditions, camera settings, complex leaf overlapping and shadowing, etc. While classical machine learning techniques such as random forest, support vector machines, and K-means clustering have proven effective for greenhouse imaging, they may encounter limitations when applied to field images [36,37,38]. To address the challenges of data analysis, numerous studies have proposed various image analysis methods, including traditional computer vision techniques and more advanced deep learning-based methods. Bai et al. applied a threshold-based method on RGB images of soybean fields to segment plants from the background and used linear discriminant analysis (LDA) and SVM models to predict the iron deficiency chlorosis (IDC) score using features extracted from plant pixels [39]. Yuan et al. adapted Otsu’s thresholding technique on early-season canopy RGB images to segment soybean from the soil background and predicted soybean end-season traits through the color and texture features [40]. Milioto et al. used a CNN with an encoder–decoder structure to perform pixel-wise semantic segmentation of crops, weeds and soil-based vegetation index and image channels of RGB field data [5]. Dash et al. utilized both unmanned aerial vehicle (UAV) and manned aircraft data to perform supervised pixel-based classification using random forest (RF) and logistic regression (LR) methods for detecting invasive conifers in a grassland environment [41]. Abeysinghe et al. assessed the effectiveness of UAV technology to identify invasive Phragmites australis using different machine learning and deep learning algorithms [42]. Neural network (NN), support vector machine (SVM) classifiers, and parametric Maximum Likelihood Classifier (MLC) on the pixel base, and K-Nearest Neighbor (kNN) on the object base were applied. Their results identified that the pixel-based NN was the best classifier for the effective eradication of Phragmites. Zhang et al. constructed a convolutional encoder neural network (CENN) to extract vegetation in farmland and woodland from Gaofen-2 remote sensing imagery [43]. These studies demonstrate the utilization of various techniques, encompassing thresholding, machine learning algorithms (SVM, LDA, RF, LR), and deep learning models (CNN, CENN), to accomplish plant segmentation for subsequent trait prediction, vegetation classification, and invasive species identification. The diverse range of methods employed indicates the capability of multiple approaches for agricultural and environmental applications. Notably, deep learning-based methods, such as CNN and CENN, are recognized as more advanced techniques in achieving accurate and efficient results in image analysis tasks within these domains.

This paper aims to address crop segmentation, one of the fundamental tasks in image semantic segmentation, with HTPP imaging data in the field using high-resolution RGB images collected by a field-based high-throughput plant phenotyping (FHTPP) platform. DCNNs have shown excellent performance in such tasks when there is a large amount of labeled image data, which usually requires a huge amount of time and labor [25,44]. With limited labeled data for training the deep learning algorithm, DCNNs tend to produce less accurate segmentation results due to the overfitting issue. For a specific FHTPP system, the images tend to be homogeneous. So, it might be sufficient to label a small number of images to get a representative training sample. Then, the actual research question is “In a relatively homogeneous population, how to adapt the deep learning algorithm to do decent semantic segmentation with a small number of training data?" Many previous researchers have focused on introducing diversification into the dataset through the implementation of various data augmentation techniques to increase data size and relieve the homogeneity [45]. In this paper, this problem is addressed by proposing a deep learning with bagging approach for semantic segmentation. Bagging is a common technique in classical machine learning, which is used to relieve the effect of overfitting and to obtain a more robust and accurate prediction. By integrating the idea of bagging into the deep learning model [46,47], we expect the model performance would be improved. The proposed framework was validated by a comparison study using different machine learning and deep learning models on an FHTTP dataset obtained from maize fields. The model training time, prediction time, and prediction IoU of thresholding, patch-to-pixel machine learning, and patch-to-patch deep learning and deep learning with bagging algorithms were compared. The results showed that the proposed deep learning with bagging algorithm has a significant improvement in segmentation accuracy. The remainder of the paper is structured as follows: the motivating dataset and the proposed deep learning with bagging approach are described in Section 2. The details of model implementation, results, and discussion are given in Section 3. Section 4 presents the conclusion.

## 2. Materials and Methods

In this section, the dataset used and data preprocessing are described in Section 2.1, and the proposed deep learning with bagging approach is presented in Section 2.2.

### 2.1. Study Area and Data Description

**Figure 1:**
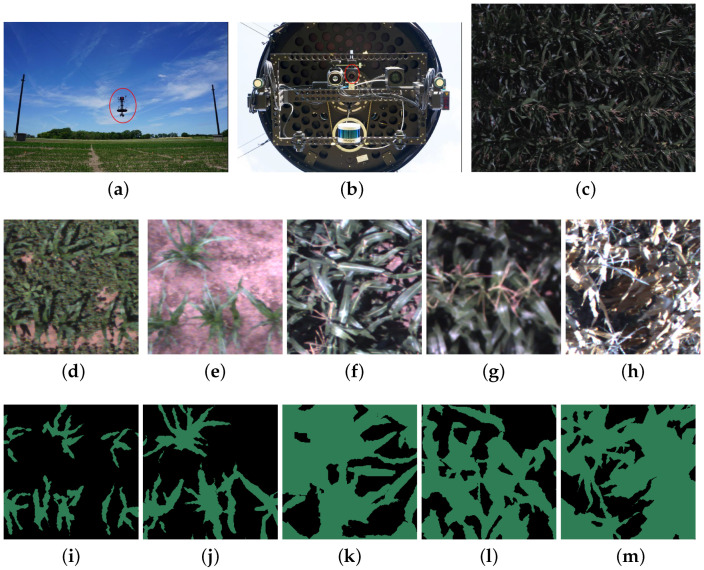
NU-Spidercam Field Plant Phenotyping Facility and the raw images used in this study. (**a**) The cable-suspended remote sensing platform (red circle). (**b**) The multispectral camera at the bottom of the sensing platform (red circle). (**c**) An example of an RGB image of a maize plot; (**d**–**h**) are example image patches and (**i**–**m**) are the corresponding labeled patches.

The maize images were taken by NU-Spidercam from June to September of 2019 and 2020. NU-Spidercam is a large-scale, integrated robotic cable-driven sensing system developed at the University of Nebraska—Lincoln as a core research facility for FHTPP research. The system comprises a cable-suspended remote sensing platform (Figure 1a), a winch control system, a subsurface drip irrigation system, an on-site weather station, and an observation building [48]. The system has been shown to be stable under windy conditions with fully automated data collection being feasible. The sensor and image data captured by the system can provide information on various aspects of plant traits such as height, ground cover, and spectral reflectance. The availability of this automated FHTPP system is expected to benefit research in field phenotyping, remote sensing, agronomy, and related disciplines. The sensing platform is designed to collect data from a 0.4 ha field and has a maximum sensor payload of 30 kg, which can be customized to integrate user-defined sensing modules. The RGB images captured by the onboard multispectral camera (AD080GE, JAI, Akishima, Kanagawa, Japan), which was installed on the Pan-Tilt structure of the platform, were utilized in this study (Figure 1b). An example RGB image captured by NU-Spidercam is shown in Figure 1c.

Semantic segmentation, which refers to assigning a label to each pixel, is one of the most important steps in many application scenarios which estimate valuable plant or canopy traits, such as leaf area index, plant density, aboveground biomass, and so on. By quantifying these traits, researchers can gain a deeper understanding of plant growth, development, and their response to environmental factors. In this study, only RGB images were used for the model development because RGB cameras are the most cost-effective and widely used ones in FHTPP. The image pixels were labeled into two classes: class 0, the background (including weed and bare soil), and class 1, the maize crop. A total of 99 full-size images were used with a spatial dimension of 1064×768 pixels. Among these images, 29 were taken in 2019 and 70 were taken in 2020. To train and validate the models, 68 images were randomly selected from the total set, while 31 images were reserved for testing. Every full-size image was cropped to 12 patches with size 256×256 pixels. Those image patches were manually examined and those with poor quality were excluded. Poor quality in certain image patches is attributed to limitations in the human labeling process, resulting in inaccurate ground-truth labels. In the end, the total numbers of training and validation patches and test patches are 750 and 328, respectively. Figure 1d–m presents several examples of these patches and their labels.

### 2.2. Methods

DCNNs are most commonly applied to process and analyze visual data, such as images or videos. They have been widely used for computer vision tasks, such as object recognition, image classification, and semantic segmentation [26,49,50]. DCNNs have shown remarkable performance in various computer vision applications, outperforming traditional machine learning algorithms and often achieving the highest accuracy rates on popular benchmarks [25]. Deeplabv3+ was adopted due to its proven effectiveness in various domains within the deep learning community [35].

This model is further combined with bagging to have more stable and accurate predictions [51,52,53]. Specifically, by training multiple deep learning estimators with different train/validation splits in parallel to capture different aspects of the data, the final prediction can be made by considering the majority vote for each pixel prediction from every estimator. This approach leverages the stochastic nature of neural network training, which can result in different local minimums of the validation loss across runs. By aggregating the predictions from multiple estimators, bagging captures this variability and reduces overfitting, and can thus generate more accurate predictions.

The following is a brief description of the Deeplabv3+ architecture. The architecture contains an encoder–decoder structure. The encoder down-samples the input images and extracts the essential information by passing images to a backbone model, a typical DCNN, and an Atrous Spatial Pyramid Pooling (ASPP) layer; whereas the decoder reconstructs the output of appropriate dimensions and makes predictions based on the information obtained from the encoder phase.

MobileNetV2 [54] was adapted as the backbone for Deeplabv3+. MobileNetV2 is a lightweight model that can run very efficiently on mobile devices without sacrificing much prediction performance. Its advantage lies in having fewer parameters compared to other architectures, resulting in efficient memory usage and faster inference, making it well-suited for resource-constrained environments, such as edge computing in domains like agricultural Internet of Things. In this specific application, characterized by relatively homogeneous image sources, limited labeled images, and limited computational resources, the lightweight nature and efficient performance of MobileNetV2 make it an ideal choice. Moreover, transfer learning was employed by leveraging pre-trained MobileNetV2 weights from the ImageNet dataset to enhance the model performance.

The loss function is a categorical cross-entropy function, which computes the cross-entropy loss between the labels and predictions. Assume the total number of label classes is *K* and each pixel is labeled as 0, 1, …, or K−1. Let $y_{i}=\left(y_{i,0},y_{i,1},\ldots ,y_{i,K-1}\right)$ be the one-hot representation of the label of the *i*th pixel; that is, if the true label class for this pixel is *k*, then $y_{i,k}$ is set to 1 and all other elements in $y_{i}$ are set to 0. The loss for one image patch is defined as

(1) $$\begin{array}{c} J\left(w\right)=-\sum_{i=1}^{H\times W}\sum_{k=0}^{K-1}y_{i,k}\log{\hat{y}}_{i,k}, \end{array}$$

where w is the model parameters, *H*, *W* are the height and width of the image patch, and ${\hat{y}}_{i}$ is the Softmax probability vector associated with $y_{i}$. The final loss is the average across all training image patches. The optimizer was Adam [55].

**Figure 2:**
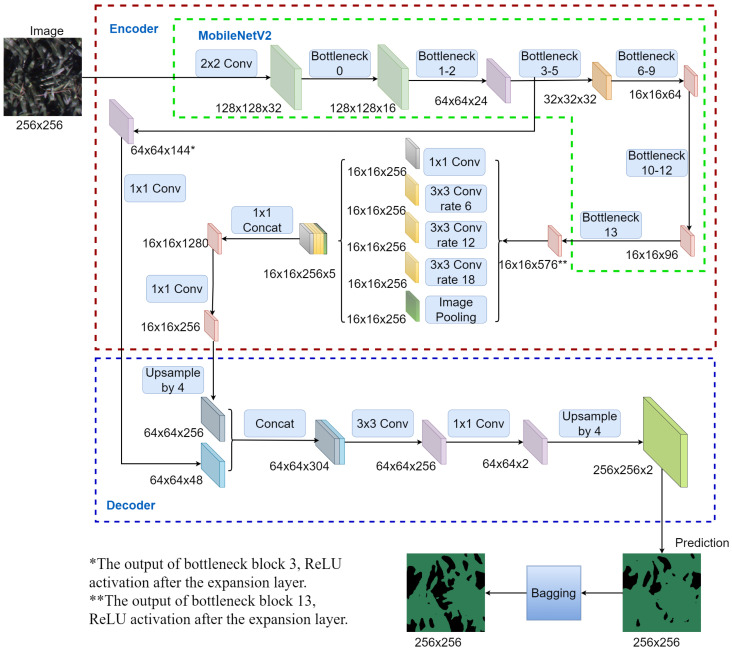
Flowchart of the proposed algorithm with the bagging approach.

The flowchart of the full algorithm with the bagging approach is shown in Figure 2.

## 3. Results and Discussion

The proposed framework was applied to the dataset described in Section 2.1. Two other semantic segmentation methods, thresholding and random forest, were implemented to evaluate the model’s performance; these are described in Section 3.1. In Section 3.2, details of the model implementation are introduced, and Section 3.3 presents the model evaluation. Additionally, the effect of the sample size used during the training stage was investigated by conducting a sequence of studies on various training and validation sizes to evaluate the trend in test performance.

### 3.1. Methods for Comparison

Thresholding is the simplest method of segmenting images. It segments images by selecting pixels with intensities within some fixed range [a,b]. To perform thresholding, the raw RGB images of maize plots were converted from RGB to Hue Saturation Value (HSV) color space; and a Hue value range covering green, yellow, and orange color spaces was used to carry out the segmentation. Certain green indices were not adopted as the threshold criteria [56] because the images were taken during the whole maize growing period and maize leaves turned substantially yellow or brown during the late stage. Focusing on just the green pixels could not segment all maize plants.

**Figure A1:**
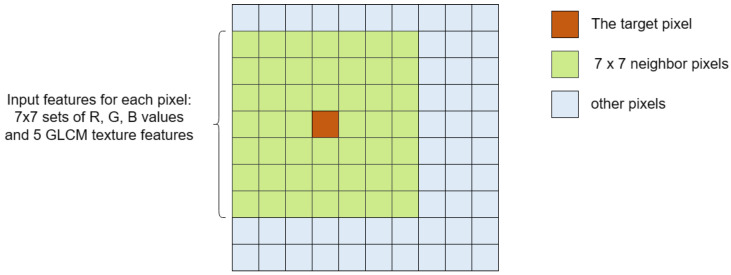
The target pixel and 7×7 neighbors centered on the target pixel.

**Figure A2:**
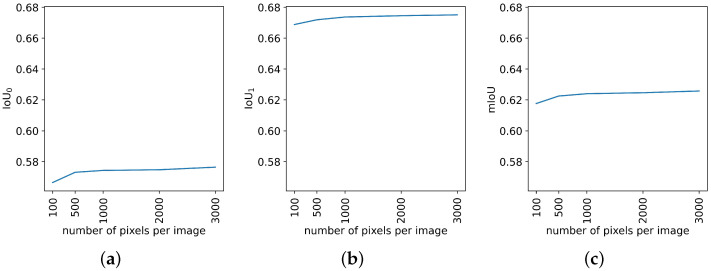
IoU curves representing the test performance of the random forest model with a 7×7 neighborhood pixel configuration across varying input numbers of pixels per image. (**a**) IoU for the plant class, (**b**) IoU for the background class, and (**c**) mean IoU across the two classes.

The random forest algorithm (RF) is an ensemble method that combines multiple decision tree predictors that each tree split depends on a random subset of the given input features [57]. It is a popular extension of bagging for decision tree models. The RF method is used for pixel-level classification in this study. The output variable represents the true label of a pixel. To incorporate the local spatial feature, R, G, B values and five texture features, contrast, dissimilarity, homogeneity, correlation, and ASM, derived on a gray-level co-occurrence matrix (GLCM) [58] of the 7×7 neighborhood pixels centered at the target pixel were utilized as the input features. For those target pixels near the border, border pixels were replicated to create 7×7 patches (Figure A1). To train the RF model, importing all the pixels from the 750 images was not feasible due to the memory restriction of the algorithm. Rather, 3000 pixels were randomly selected from each image and 2,250,000 obtained pixels as model input. Note that our experiment with fewer data showed that increasing the number of sampled pixels will not lead to a significant improvement in the model’s performance, given the diminishing returns associated with additional data (Figure A2). This suggests that the current dataset provides a representative and sufficient amount of information for training the RF model.

### 3.2. Model Implementation

All the algorithms described above were implemented at the Holland Computing Center (HCC) at the University of Nebraska—Lincoln. The configurations of the HCC cluster used in this study were RAM of 32 GB and two GPUs of NVIDIA Tesla V100 with 32 GB memory (Santa Clara, CA, USA). The same training, validation, and test datasets were used to gain fair comparisons.

The algorithms were fine-tuned with different hyperparameter settings to achieve the highest performance. For the thresholding, a grid search of the hue range [a,b] was implemented to segment plant pixels, with *a* ranging from 0 to 40 and *b* ranging from 80 to 130 with both intervals of 10; all the *a*, *b* combinations were tested. The total sum of squared error (SSE) was calculated for each combination, and the one with the least SSE was selected. The final range used was [10, 110].

For the random forest algorithm, two hyperparameters were fine-tuned in five-fold cross-validation based on mean cross-validated accuracy. They are the maximum number of depths in each decision tree (ranging from 10 to 50 with an interval of 10) and the number of trees in the forest (ranging from 50 to 200 with an interval of 50). Other hyperparameters keep the default value of the random forest classifier from the sklearn library in Python 3.6. The final choices of hyperparameters are 50 depths and 200 trees.

For the deep learning algorithm, batch sizes (4, 8, 16, 32), learning rates (0.01, 0.001, 0.0001) and dropout rates (ranging from 0.1 to 0.9 with an interval of 0.1) were fine-tuned based on validation loss. All 750 image patches were used, among which 600 were training patches and 150 were validation patches. Deep learning models ran 50 epochs with a total of 4000 training images per epoch from image augmentation on the training set. The augmentation methods included random rotation, shifting, flipping, shearing, and zooming. The chosen learning rate, batch size, and dropout rate were 0.001, 8, and 0.5, respectively, and the default recommended values $\beta_{1}=0.9$, the exponential decay rate for the first moment estimates, and $\beta_{2}=0.999$, the exponential decay rate for the second moment estimates for the Adam optimizer were used. The best weights that led to the smallest validation errors were saved for predicting and calculating the IoU on the test dataset.

Finally, because of the homogeneity of the field and plant species, as well as a relatively small dataset for deep learning, the model was repeated 60 times in parallel on the HCC cluster to explore the effect of bagging by taking the majority vote for each pixel given the predicted image patches.

### 3.3. Model Performance

**Figure 3:**
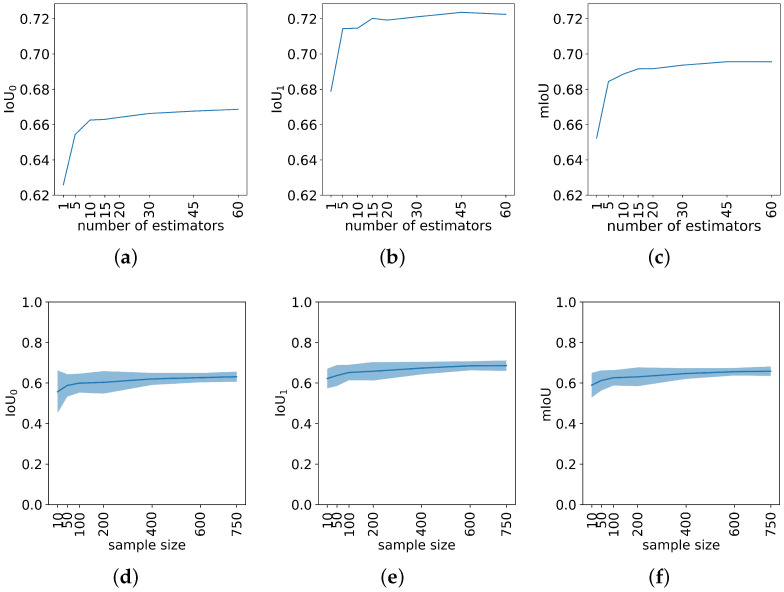
(**a**–**c**) IoU curves for the plant class, background class, and the mean IoU across both classes in the test data set. These curves are generated from the bagging models with varying numbers of estimators. (**d**–**f**) Mean curves and their 95% confidence bands (mean ± t × standard deviation) of IoUs for the plant class, background class, and the mean IoU across both classes in the test data set.

The prediction results were quantified by Intersection-over-Union (IoU), also known as the Jaccard index, which is defined as the area of overlap divided by the area of the union of the predicted region and the ground-truth region. It is a good metric for measuring the similarity between the two regions and can take into account the class imbalance issue [59]. The IoU value ranges from 0 to 1, where 0 indicates no overlap between the predicted and ground-truth regions, and 1 indicates a perfect match between the two regions. $IoU_{i}$ was defined as the IoU for class *i*, where i=0,1, and mean IoU (mIoU) as the arithmetic mean over the two classes. Figure 3a–c show the predicted IoU increases when the number of estimators, which refers to individual CNN models, increases from 1 to 60. The steepest ascent occurs at the interval [1, 10], then the performance becomes stable after 45 estimators. Overall, bagging performs universally better and it brings another 7.7% improvement in mIoU, from 0.65 to 0.70, which demonstrates the effectiveness of bagging.

Table 1 summarizes the training time ($t_{1}$), the prediction time for one image patch ($t_{2}$), the IoUs for class 0 and class 1, and the mean IoU on the test patch set for each method.

As shown in Table 1, the threshold model does not require training and sets a fixed threshold to classify objects, resulting in an mIoU of 0.50. The RF model achieves an improved mIoU of 0.63 after 13.5 h of training with a relatively fast detection speed of 8.5 s per prediction image. The Deeplabv3+ model achieves an even higher mIoU of 0.65 with a faster training time of less than 2 h, but with a slightly slower detection speed of 0.06 s per prediction. However, it is worth noting that the Deeplabv3+ model did not show improved performance compared with RF on $IoU_{1}$. They both achieved a 0.68 $IoU_{1}$ score.

Moreover, to further improve the Deeplabv3+ model’s performance, the Deeplabv3+ model with bagging is trained with multiple estimators, each taking less than 2 h to train. As a result, it achieves the highest mIoU of 0.70 among all models with 45 estimators, at the cost of computational resources.

Overall, CNN-based deep learning algorithms achieved higher IoU than RF and thresholding, especially in the background class (class 0). This is because the convolution operations in CNN are performed on the 2D neighboring pixels that can effectively extract local spatial features. These local spatial features are then combined to form higher-level features by going through multiple convolutional layers. Yet, RF only includes neighborhood pixels to account for spatial dependence among image pixels, which leads to poor predictions. Thresholding has the lowest performance. Note that traditional methods are still accurate for simple images such as greenhouse images. However, when more complex segmentation tasks are needed, e.g., plant segmentation of natural field images, the deep learning-based algorithms are much more useful. The complexity of field images is not only from the variation in plants (growing stages, color, size) and the background (soil, green weed, crop residue), but also from the variation in natural illumination (sunny vs. cloudy days, sun-lit vs. shaded parts of an image), etc.

On the other hand, Deeplabv3+ needs less training and prediction time than RF. This is because the RF method here is a patch-to-pixel prediction algorithm. To predict the whole image patch with resolution 256×256, the prediction needs to run $256^{2}$ = 65,536 times. For instance, the RF method takes 2.5 h to predict 1000 image patches while the proposed patch-to-patch deep learning models just need around 1 min. Thus, RF is not practical in real-world applications where the prediction time is one of the major concerns. Though thresholding seems the most efficient, the low prediction IoU would be an issue.

**Figure 4:**
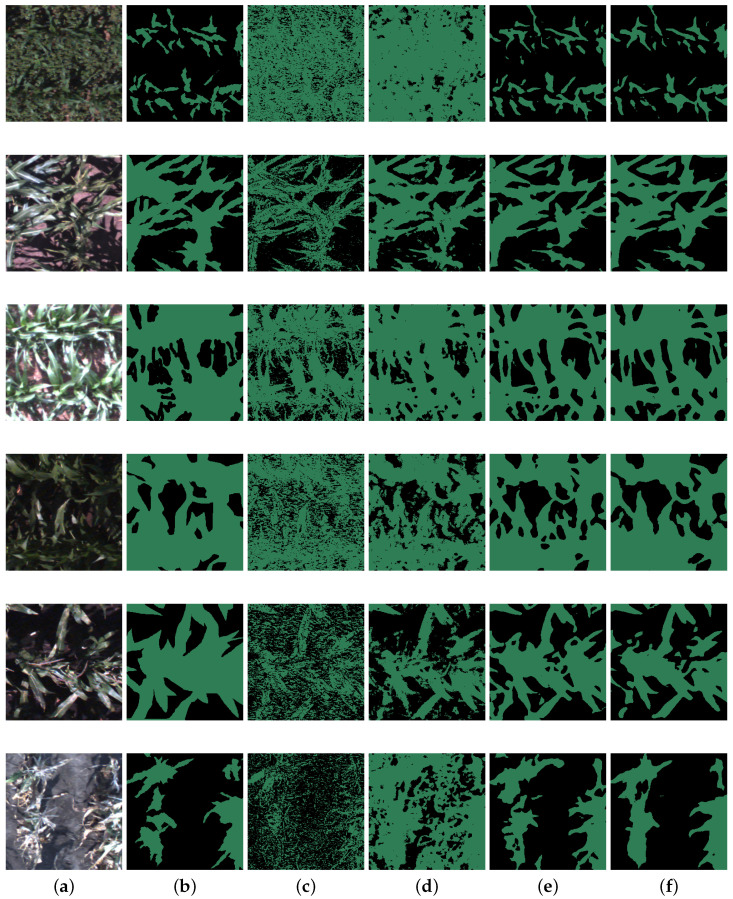
A visualization model prediction performance. (**a**) RGB patches; (**b**) labeled patches of (**a**); (**c**) thresholding prediction of (**a**); (**d**) RF prediction of (**a**); (**e**) Deeplabv3+ prediction of (**a**); (**f**) Deeplabv3+ with bagging prediction of (**a**).

Figure 4a–f illustrate example RGB image patches, labeled patches, and the corresponding prediction patches for various growing stages and conditions. The patches are arranged in temporal order from the plant emergence stage to the maturity stage. Groups (e) and (f) represent results from deep learning-based algorithms. These two groups are more closely aligned to group (b), the ground truth, compared with groups (c) and (d), which are obtained from conventional machine learning algorithms. Specifically, the topmost image patch shows visible weeds in the background as maize plants are small during their early stage. Deeplabv3+ is the only algorithm capable of classifying weed pixels into the background class. The second and third patches were captured on sunny days with ideal illumination, showing some parts that are sun-lit and some parts that are shaded. The fourth and fifth patches were taken on cloudy days with most parts covered by shade. Deep learning models perform better under both conditions. Lastly, in the bottom image patch, maize leaves are yellowing, making it harder to distinguish them from the soil. Neither thresholding nor RF produces a satisfactory classification, whereas the Deeplabv3+ models capture the yellow leaf feature and make an ideal prediction. While comparing the standalone Deeplabv3+ model to the Deeplabv3+ with bagging model, the latter approach exhibits even higher accuracy and robustness in plant semantic segmentations. This improvement can be attributed to the utilization of ensemble learning, which allows the model to leverage the collective knowledge of multiple individual models. Based on these findings, it is recommended to prioritize the deep learning with bagging method over the standalone deep learning algorithms for plant semantic segmentation, provided that sufficient computational resources are available during the training stage. In conclusion, the results demonstrate that the deep learning algorithms perform successful segmentation for all field images across environments, and they operate efficiently in a patch-to-patch manner.

Additionally, to evaluate the number of labeled images required for having decent prediction accuracy in semantic segmentation, a sequence of standalone Deeplabv3+ models were trained on increasing sample sizes. In each case, 80% of the samples were used for training and the remaining 20% for validation. This process was repeated 30 times for each sample size, with different random seeds for image selection and train–validation splits, to obtain the mean IoU curve and confidence band. As shown in Figure 3d–f, the prediction mIoU increases by only around 8% when the training sample size increases from 10 to the maximum 750, due to the binary segmentation objective and relatively homogeneous field. The steepest increase occurs in the interval of [10, 100], and the performance becomes stable after 400. Thus, deep learning algorithms require a much smaller labeled sample set during the training stage to achieve satisfactory test performance, which efficiently reduces the labeling time, effort, and budget.

Given the strong performance of this methodology and the significance of crop segmentation, we believe it could be applied to various crop types with a limited number of available labels. Furthermore, integrating this model into a stacked processing pipeline can enhance the delivery of more parameters for precise field management. These parameters may include weed and insect detection and localization, growth stage estimation, and yield prediction.

## 4. Conclusions

In this paper, a deep learning and bagging algorithm was proposed on high-resolution RGB images collected in various illumination and field conditions by an HTPP system for semantic segmentation due to its relative homogeneity and scarce resources for pixel-wise labeling. Thresholding, random forest, a standalone deep learning model, and the deep learning model with bagging were evaluated using the NU-Spidercam dataset. The evaluation compares their model training time, prediction time, and prediction IoUs.

In many real-world applications of machine learning, the primary objective is often to make accurate predictions to segment objects, predict future trends or identify classes. The end-users rely on the prediction results to make data-driven decisions often prioritizing the prediction accuracy over aspects related to the model training process, such as training accuracy and time. Our findings demonstrate the effectiveness of deep learning models, as they outperform the other algorithms with a prediction mIoU of 0.65 and require less training and prediction time compared to the random forest model. Additionally, the deep learning model with bagging further improves prediction performance by 7.7% to a mIoU of 0.70, leveraging the power of ensemble learning. However, it is worth noting that this improvement comes at the cost of increased computational resources during the training stage. While thresholding stands out as the most efficient algorithm, its lower prediction performance, which is 29% lower than the proposed bagging approach, renders it impractical for real-world applications. Therefore, deep learning with bagging is identified as the most suitable choice among the three types of algorithms, considering that prediction performance is of utmost importance in practical scenarios. Furthermore, the deep learning with bagging model showcases its capability of handling the complexities of Spidercam data, such as variations in lighting and background conditions, which significantly impact segmentation accuracy. By leveraging the power of deep learning and bagging, this approach overcomes the limitations of conventional segmentation methods and achieves superior performance in challenging scenarios.

Computer vision tasks are expected to increasingly rely on deep learning models in the near future. One unique issue preventing the applications of AI in similar studies is the small set of labeled images as the labeling costs can be particularly high. In this study, the effect of sample size on the deep learning model was also investigated. The results revealed that a smaller labeled sample set, specifically 400 out of 750 training patches, would be adequate for the segmentation tasks examined here, as the mean prediction IoU curves reached a plateau shortly after the sample size increased. This study demonstrates this label anxiety can be mitigated to a certain degree. Such findings can help guide the design and implementation of future phenotyping studies using high-resolution RGB images, enabling researchers to achieve satisfactory prediction performance with a smaller labeled sample set. Additionally, given the strong performance of this methodology and the significance of crop segmentation, we believe it could be applied to various crop types with a limited number of available labels. Furthermore, with the continuous improvement of the spatial resolution of remote sensing images, the proposed data processing pipeline for efficient semantic segmentation, encompassing the deep learning and bagging approach, has the potential to become a valuable approach in a more general scenario to enhance the delivery of more parameters for precise field management such as weed and insect detection, and localization, growth stage estimation, and yield prediction.

## Figures and Tables

**Table 1:** The performance evaluation of different models.

Model	$t_{1}$	$t_{2}$	${\mathrm{IoU}}_{0}$	${\mathrm{IoU}}_{1}$	mIoU
Threshold	NA	0.02 s	0.46	0.54	0.50
RF	15 h	7 s	0.58	0.68	0.63
Deeplabv3+	<2 h	0.06 s	0.62	0.68	0.65
Deeplabv3+ (bagging) *	<2 h per estimator	0.06 s per estimator	0.67	0.72	0.70

* Forty-five estimators were used for the bagging Deeplabv3+ result.

## Data Availability

The paper is accompanied by publicly available source code at https://github.com/yzasdfg/Deep-Learning-and-Bagging-for-Efficient-Semantic-Segmentation-in-Spidercam-Plant-Phenotyping-Studies accessed on 13 April 2024.
